# Epicardial infrared ablation to create a linear conduction block on a beating right atrium

**DOI:** 10.1186/s13019-018-0801-y

**Published:** 2018-11-16

**Authors:** Hiroshi Kubota, Hidehito Endo, Hikaru Ishii, Hiroshi Tsuchiya, Yusuke Inaba, Yu Takahashi, Katsunari Terakawa

**Affiliations:** Department of Cardiovascular Surgery, Kyorin University, 6-20-2, Shinkawa, Mitaka, Tokyo, 181-8611 Japan

**Keywords:** Atrial fibrillation, Ablation, Coagulator, Energy source, Electrophysiology, Arrhythmia treatment, Infrared, Minimally invasive surgery, Maze procedure, Photocoagulation

## Abstract

**Background:**

It is still difficult to create a secure linear conduction block on a beating heart from the epicardial side. To overcome this drawback we developed an infrared coagulator equipped with a cuboid light-guiding quartz rod. This study was designed to electrophysiologically confirm the efficacy of a new ablation probe using infrared energy in a clinical case.

**Methods:**

The infrared light from a lamp is focused into the newly developed cuboid quartz rod, which has a rectangular distal exit-plane that allows 30 mm × 10 mm linear photocoagulation. Two pairs of electrodes were attached to the right atrium of a patient who was undergoing surgery. Each pair of electrodes was placed 10 mm from an ablation line. The change in conduction time between the two pairs of electrodes was measured during ablation. The predicted conduction time delay ratio was 1.54.

**Results:**

The actual conduction time after ablation was 1.38–1.43 times longer than the pre-ablation conduction time.

**Conclusions:**

The infrared ablation using a newly developed cuboid probe made it possible to create a linear conduction block on the beating right atrial free wall clinically.

## Background

**Fig. 1:**
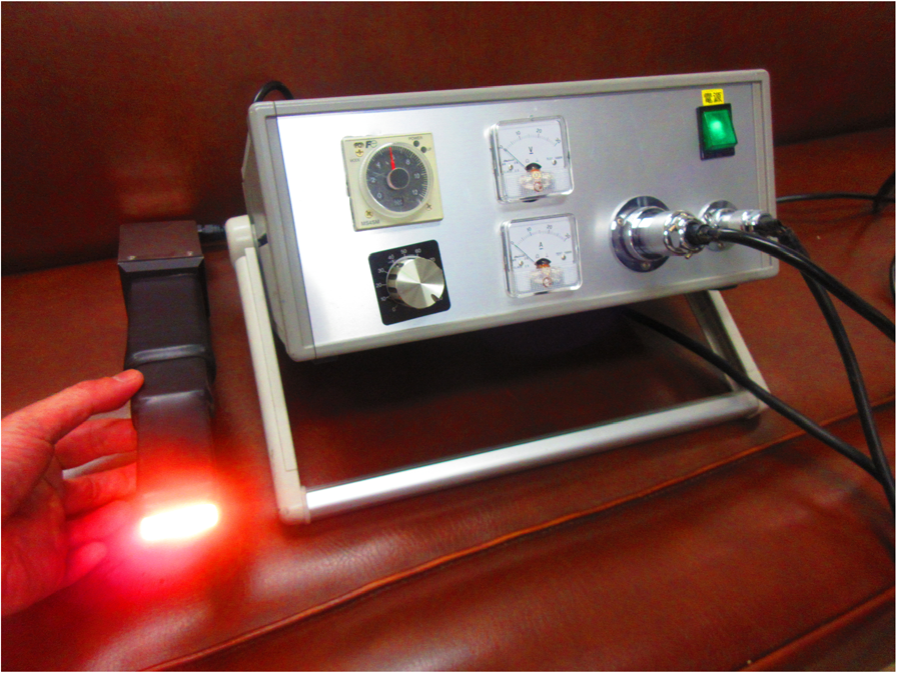
Infrared coagulator “Kyo-co”. The cuboid quartz rod exit plane has a rectangular (30 mm × 10 mm) surface designed to enable creation of a linear lesion. Light from a tungsten-halogen lamp emerges as 35 W/cm^2^ of near-infrared light energy (wavelength: 400 to approximately 1600 nm; peak wavelength: 850 nm)

The newly developed infrared coagulator, named the “Kyo-Co (Photon incorporation, Saitama, Japan)”, contains a reflector that focuses light from a tungsten-halogen lamp into a light-conducting 30 mm × 10 mm cuboid quartz rod, and the light emerges as 35 W/cm^2^ of near-infrared light energy (wavelength: 400 nm to approximately 1600 nm; peak wavelength: 850 nm). The distal exit-plane of the light-conducting rod has a rectangular plane surface (30 × 10 mm) (Fig. 1).

## Methods

In a preliminary experiment, the probe applied to a specimen of chicken muscle tissue, and the muscle tissue was ablated for a total of 28 s (4 s × 5 times at 2 s intervals) (*n* = 5). Tissue temperature with time was measured with a thermometer Ti480® (Fluke Corporation, WA, U.S.A.). The maximum temperature of muscle tissue was measured. After ablation of the chicken muscle the depth of the lesion was measured macroscopically.

**Fig. 2:**
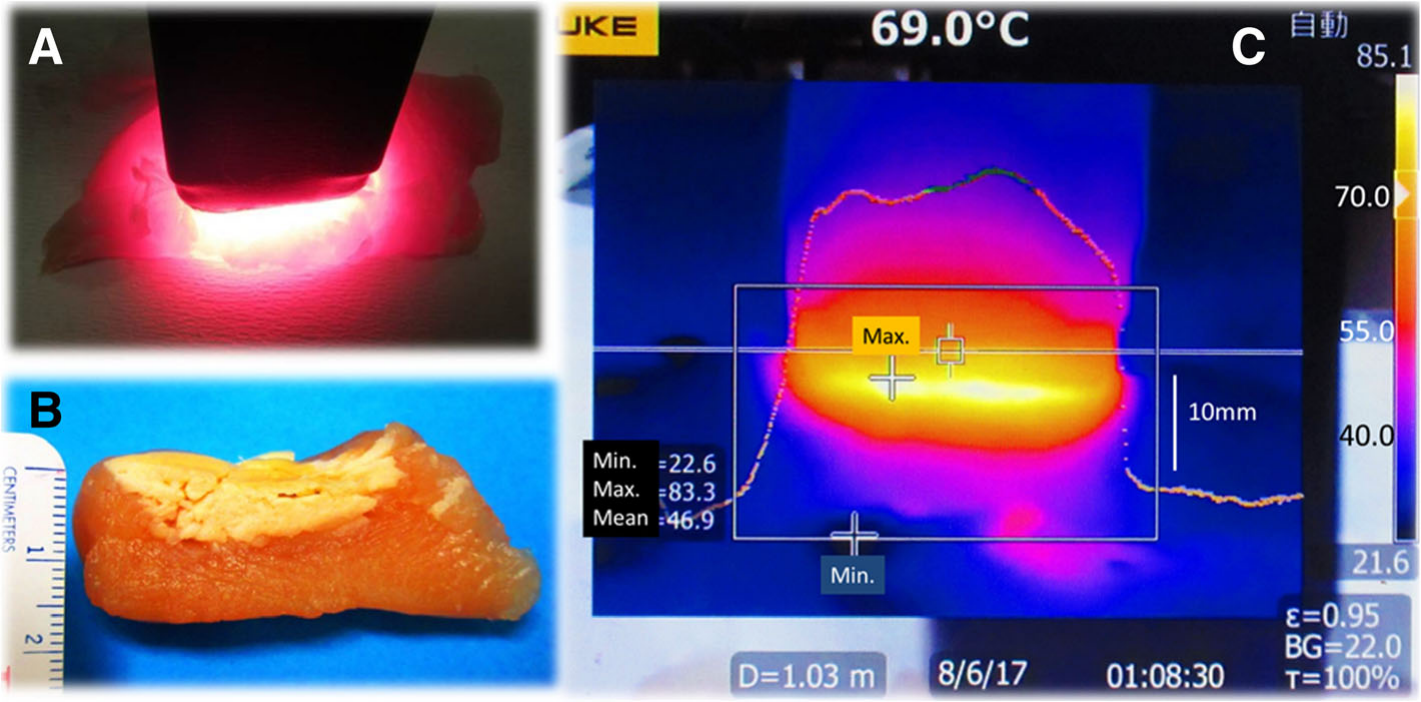
Preliminary experiment on chicken muscle tissue. **a** The probe was pressed against the muscle tissue. **b** The mean depth of the lesion was 8.7 + 0.8 mm. **c** The maximum temperature was 97.9 + 2.1 °C

The maximum temperature of the chicken muscle was 97.9 + 2.1 °C, and the depth of the lesion was 8.7 + 0.8 mm (Fig. 2).

### Clinical experience

In 2014, the ethics committee of Kyorin University approved a clinical and epidemiologic study entitled, Surgical treatment of arrhythmias, infectious endocarditis, infected aortic aneurysms, and cardiac tumors with an infrared coagulator. Written consent was obtained from the patient.

**Fig. 3:**
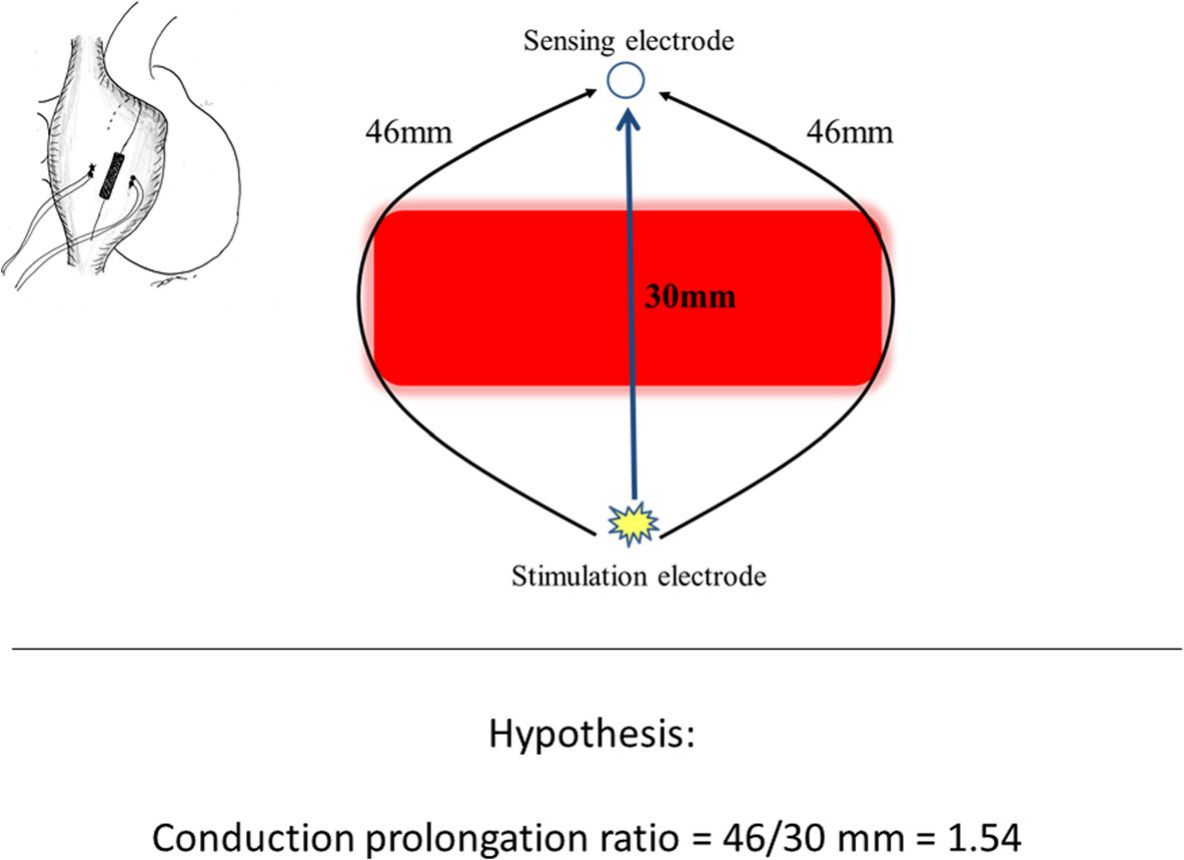
Prediction. Because the conduction distance prolongation ratio is directly proportional to the conduction time prolongation ratio, when a linear transmural lesion is created in the same shape as the rectangular exit plane, the conduction time prolongation ratio is predicted to be 1.54

We hypothesized that when a rectangular transmural lesion is created in the same shape as the exit plane of the cuboid quartz rod, the translesion conduction time would be prolonged. We predicted that the conduction time prolongation ratio (post-ablation conduction time/pre-ablation conduction time) would be directly proportional to the conduction distance prolongation ratio (post-ablation conduction distance/pre-ablation conduction distance). The predicted conduction prolongation ratio was 46.1/30.0 mm = 1.54 (Fig. 3). After obtaining written informed consent from the patient, in August 2016 mitral and tricuspid valve plasty and a maze procedure were performed on a 64-year-old man with infectious endocarditis, severe mitral regurgitation, moderate tricuspid regurgitation, and paroxysmal atrial fibrillation. After a median sternotomy, the pericardium was opened, and the epicardial atrial ablation and electrophysiological study were performed before commencing the cardiopulmonary bypass. Two pairs of alligator clip electrodes were attached to the right atrium 10 mm from the expected ablation line. The pair of electrodes attached on the dorsal side of the ablation line was used to pace the right atrium, and the pair of electrodes attached on the ventral side of the ablation line was used as sensing electrodes to record the atrial potential. The cuboid 30 mm long 10 mm wide quartz rod of the infrared coagulator was applied epicardially to create a linear lesion on the free wall of the beating right atrial free wall as part of the incision line of the maze procedure (Fig. 3). The total duration of each ablation was 28 s applied in a 5 series of 4.0 s each at 2.0 s intervals. The pacing rate was set to 90 bpm. An electrocardiogram (ECG) and atrial potentials were recorded with an HPM 4500 polygraph (Fukuda Denshi, Tokyo, Japan).

**Fig. 4:**
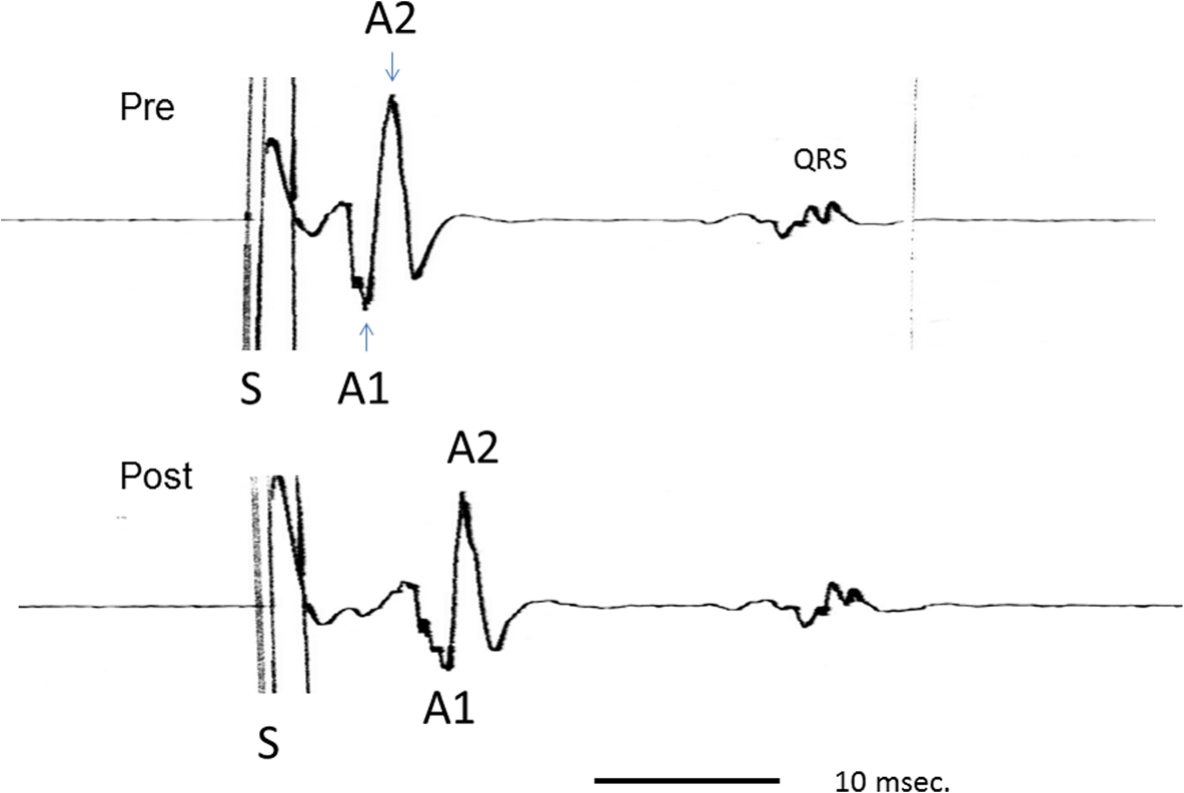
Atrial potential pre- and post-ablation. The atrial potential was biphasic in shape. Conduction times were measured as the interval between stimulation (S) and each peak of the atrial potential (A1 and A2)

Because the atrial potential was biphasic in shape, conduction times were measured as the interval between stimulation (S) and each peak of the atrial potential (A1 and A2, Fig. 4), and plotted. After the experiment, a square specimen with a side length of 5 mm of coagulated right atrial wall was excised, stained with Masson trichrome, and examined microscopically.

## Results

**Fig. 5:**
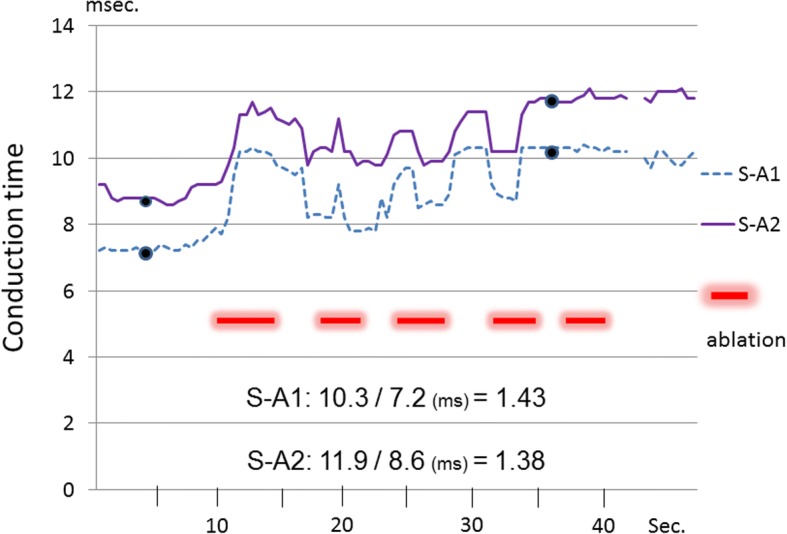
Atrial potentials pre-ablation and post- ablation (S: stimulation). Biphasic atrial potentials were recorded (A1 and A2). Both S-A1 conduction time and S-A2 conduction time were prolonged after the 28 s of ablation, and the conduction prolongation ratios were 1.43 and 1.38, respectively

From 1st to 4th infrared application, both S-A1 conduction time and S-A2 conduction time were prolonged and incompletely reversed each time (Fig. 5).

After 4th infrared application, both S-A1 and S-A2 conduction time plateaued, and the 5th infrared application did not affect either conduction time.

S-A1 conduction time was prolonged from 7.2 ms to 10.3 ms. The conduction prolongation ratio was 10.3/7.2 = 1.43.

S-A2 conduction time was prolonged from 8.6 ms to 11.9 ms. The conduction prolongation ratio was 11.9/8.6 = 1.38.

**Fig. 6:**
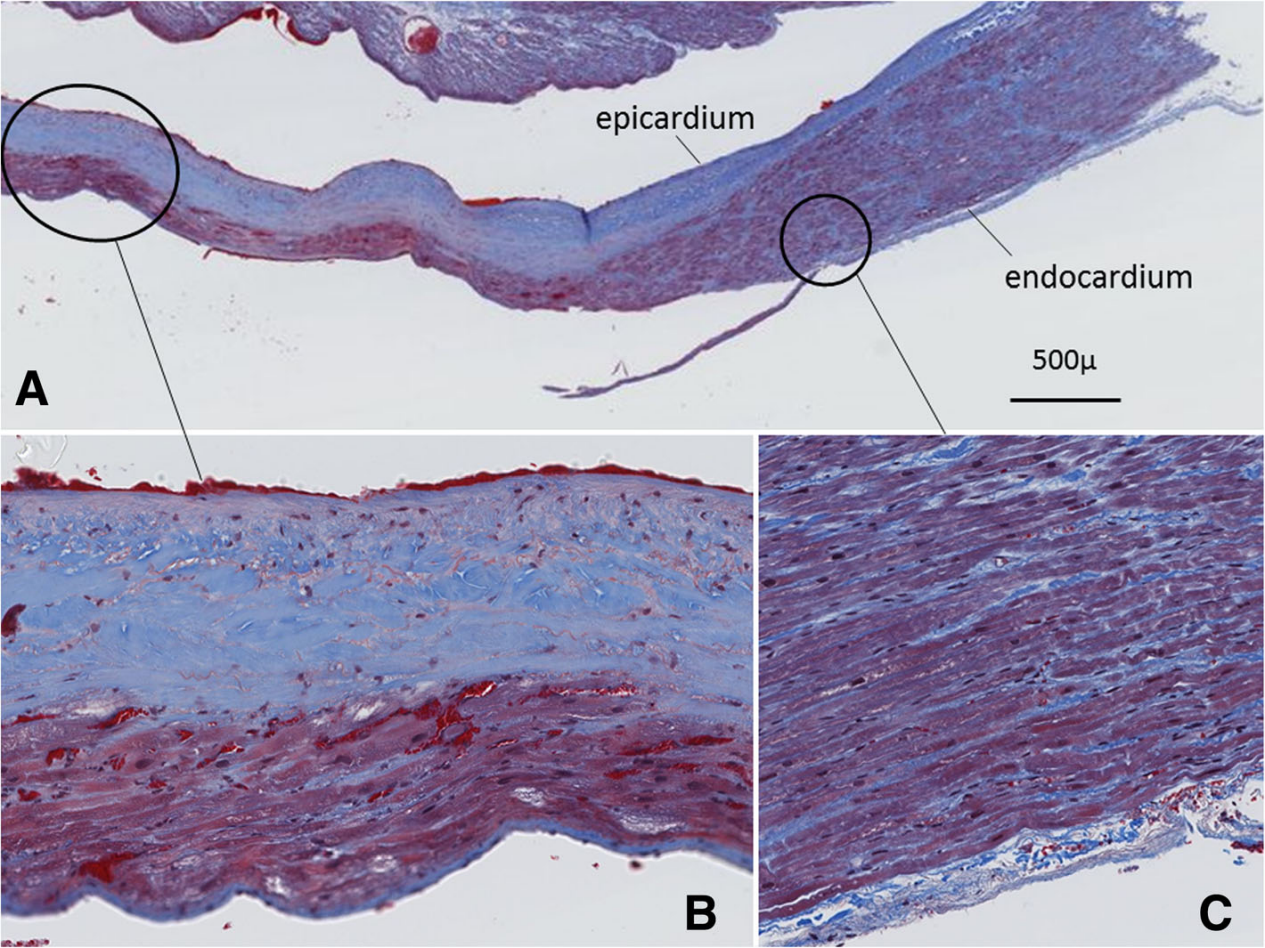
Histopathologic changes in the ablated right atrium. **a** Histologic examination showed preservation of both the endocardium and epicardium of the coagulated lesion. Both the endocardium and the epicardium were intact. **b** Severely degenerated myocardium was observed from the epicardial side to the mid-portion of the atrial wall. The endocardial side showed swelling and hyperchromatosis of the nuclei, acidophilic change in the cytoplasm, and deformity of the myocardium. **c** Myocardium was intact at the endocardial side of the marginal legion

Histopathologic examination of the ablated right atrium showed preservation of both the endocardium and epicardium of the coagulated lesion (Fig. 6). Severely degenerated myocardium was observed from the epicardial side to mid-portion of the atrial wall. Swelling and hyperchromatosis of the nuclei, acidophilic change in the cytoplasm, and deformity of the myocardium were observed in the myocardium on the endocardial side.

## Discussion

To realize the epicardial maze procedure, the major drawback is how to make the transmural lesion on the beating atrial free wall under the condition of existence of inner warm blood flow which weakens heating/cooling effect of the ablation device.

Nath et al. demonstrated that hyperthermia induced by radiofrequency energy causes significant changes in the electrophysiological properties of myocardiocytes, including membrane depolarization, reversible and irreversible loss of excitability, and abnormal automaticity, in an in vitro isolated guinea pig right ventricular papillary muscle model. They observed reversible loss of cellular excitability and tissue injury after exposure to temperatures in the 42.7 °C to 51.3 °C range (median, 48.0 °C) for 60 s and irreversible loss of cellular excitability and tissue injury after exposure to temperatures > 50 °C for 60 seconds [1].

Bulava et al. assessed the efficacy of epicardially created lesions produced with bipolar radiofrequency (RF) energy in 70 patients who had persistent, longstanding atrial fibrillation [2] and reported achieving complete isolation of the posterior left atrial wall in only 22.9% of the patients. The success rates for creating conduction block across the inferior pulmonary veins (PVs) and across the roof line connecting the two superior PVs were only 58.0 and 24.3%, respectively. Right PVs were found to have been isolated in a significantly higher proportion of patients (91.4%) than the left PVs (75.7%) were. The low efficacy of epicardial RF ablations in creating a transmural irreversible electrophysiological block, especially on the “unclampable free wall” of the atrium, represent that heat sinking effect of the inner blood flow that weaken the thermal effect of the RF.

We previously reported the fundamental results of using an infrared coagulator in animal models [3–5]. The results of the series of experiments in animal models revealed that an infrared coagulator enables creation of a transmural lesion on the canine beating right ventricle to a maximum depth of 10.3 mm, a conduction block on an arrested heart, and a conduction block on a beating right atrium. A successful epicardial maze procedure and successful electrical isolation of the right atrial appendage with an infrared coagulator and concomitant on-pump beating coronary artery bypass grafting have been reported in a clinical case [6, 7].

A cuboid probe was newly developed to create a linear lesion and conduction block on the atrial free wall. To demonstrate the conduction block on the beating heart, encircled lesions e.g. the both atrial appendage, PV cuffs, or box lesion are easy because the ineffective overdrive pacing from inside the lesion can prove the exit block. By contrast, it is difficult to demonstrate a conduction block on a beating atrial free wall. Intraoperative epicardial mapping with multi-electrodes is in common use, but it requires a large-scaled mapping system. Furthermore, because the multi-electrodes are just placed on the epicardium, not attached to it, they slip, and it is difficult to be estimate the exact electrical conduction time. A polygraph with two channels was used in our patient, and it was possible to measure the exact conduction time in every heart beats. We hypothesized that an increase in the translesion stimulus-excitation delay indicates a continuous, transmural, linear lesion, and that the translesion stimulus-excitation delay is directly proportional to path length in the viable tissue around the lesion. Himel et al. demonstrated that complete lesions with RF in rabbit hearts increase translesion stimulus-excitation delay, whereas incomplete lesions do not increase the delay [8]. As far as we investigated, there is no report that proved the stimulus excitation delay on the ablated epicardial atrial free wall in a clinical case. The right atrial free wall was selected for examination. It is easy to apply the cuboid-shaped probe to the right atrial free wall, and it was thought that it would be more difficult to make a transmural lesion in the right atrium than in the left atrium, because the right atrium has a complicated inner structure due to its thick trabecular muscle.

In our patient the myocardium was ablated intermittently at 2-s intervals. Intermittent ablations are more effective than long continuous ablations, because the intervals prevent rapid temperature rise, and prevent to make charring that blocks the photo-energy radiation deep inside the myocardium.

The actual conduction prolongation ratios in the present study were smaller than the predicted ratios, and the main reason for the smaller ratios is thought to be that it was difficult to determine the exact distance between the ablation line and alligator clip electrodes. To determine the more acculate distance, using small bipolar needle electrodes is better. Marginal non-transmural lesions and atrial tissue shrinkage are considered to be other factors that affect conduction time; both factors may shorten the conduction distance and reduce the prolongation ratio.

Pathological examination of the tissue obtained from our patient confirmed the presence of transmural degeneration, however, it was not homogeneous. It may represent the heat sinking effect of the inner blood flow. Considering the result of the presented electrophysiologocal study, there may be a discrepancy between the histopathological transmurality of the lesion and its electrophysiological transmurality. The histopathological change may not exactly represent the electrophysiological change but instead underestimate it by overlooking the pathologically normal but electrophysiologically remodeled lesion of the myocardium. Chronic reversibility of the prolonged conduction time was not investigated in this study. However, in a previous animal study we verified hemosiderin deposition, macrophage invasion, increased capillary vessels, and increased juvenile elastic fibers in the right atrial free wall 3 months after ablation. The myocardium did not revive, the endocardium became thickened, and elastic fibers appeared.

Theoretically, same as RF, this new technology can be applied not only to the atrium but also to the ventricle. Modifying the shape and flexibility of the probe may enable safe and effective minimally invasive endoscopic ablation to create a box lesion on the beating left atrium and to treat ventricular tachycardia based on the same tissue photocoagulation principle as described above.

## Conclusions

The newly developed cuboid probe of the Kyo-co infrared coagulator may have the potential to serve as a reliable device for performing the epicardial maze procedure on the beating atrial free wall clinically.

## Abbreviations

ECG: Electrocardiogram
RF: Radiofrequency
